# East Asian *Mycobacterium avium* subsp. *paratuberculosis*: molecular typing and population structure

**DOI:** 10.4142/jvs.26058

**Published:** 2026-06-05

**Authors:** Jun Ho Lee, Su Min Kyung, Eun-Seo Lee, Han Gyu Lee, Eun-Yeong Bok, Srinand Sreevatsan, Han Sang Yoo

**Affiliations:** 1 Department of Infectious Disease, College of Veterinary Medicine, Seoul National University, Seoul 08826, Korea.; 2 BK21 FOUR Future Veterinary Medicine Leading Education and Research Center, College of Veterinary Medicine, Seoul National University, Seoul 08826, Korea.; 3 Research Institute for Veterinary Science, College of Veterinary Medicine, Seoul National University, Seoul 08826, Korea.; 4 Division of Animal Diseases & Health, National Institute of Animal Science, Rural Development Administration, Wanju 55365, Korea.; 5 Department of Pathobiology and Integrative Biomedical Sciences, College of Veterinary Medicine, University of Missouri, Colombia, MO 65211, USA.

**Keywords:** Molecular epidemiology, East Asia, paratuberculosis, phylogeny, one health

## Abstract

**Importance:**

The global molecular epidemiology of *Mycobacterium avium* subsp. *paratuberculosis* (MAP) has been well documented in North America and Europe, but the population-genetic framework of the East Asian lineages is underrepresented, hindering a complete understanding of the global genotype distribution and transmission dynamics.

**Objective:**

This study aimed to establish a regional molecular-typing framework for MAP in East Asia and investigate its genetic relatedness with global lineages to explore potential intercontinental connections associated with international livestock movements.

**Methods:**

The study integrated 12 newly characterized MAP isolates from South Korean ruminant herds (cattle and black goats, 2021–2024) with 34 global isolates across six continents. Genotypic characterization was performed using mycobacterial interspersed repetitive unit-variable number tandem repeat (MIRU-VNTR) and multilocus short sequence repeat (MLSSR) approaches to evaluate the allelic diversity and subtyping resolution.

**Results:**

All South Korean isolates were identified as “cattle-type” (Type II) and predominantly belonged to the INRA Nouzilly MIRU-VNTR (INMV) genotype. Age-associated differences in MAP prevalence were observed among Korean ruminant herds (Fisher’s exact test, *p* < 0.05), with distinct age-related patterns in beef and dairy cattle The integrated MIRU-VNTR and MLSSR approaches identified 14 and 31 distinct subtypes, respectively, with MLSSR showing superior discriminatory power (discriminatory index = 0.973 vs. 0.630 for MIRU-VNTR). This high allelic diversity in the global MLSSR dataset provided finer resolution than MIRU-VNTR for distinguishing the MAP subtypes across continents.

**Conclusions and Relevance:**

This study provides a reference point for understanding the global diversity and distribution of MAP by bridging the genomic information gap in Asia. These findings underscore the potential of high-resolution, MLSSR-integrated surveillance as a practical tool for monitoring MAP across diverse geographical scales and for prioritizing isolates for future whole genome sequencing-based studies.

## INTRODUCTION

Johne’s disease is a chronic granulomatous enteritis affecting primarily ruminants, caused by *Mycobacterium avium* subsp. *paratuberculosis* (MAP). In advanced clinical states, the disease manifests as persistent diarrhea, weight loss, and a protein-losing enteropathy, eventually leading to death [1]. The disease is distributed globally and leads to substantial economic losses in the cattle industry through premature culling and reduced productivity [2]. In addition, concerns regarding the potential association between MAP and Crohn’s disease in humans have emerged, prompting discussions about food safety [3]. Nevertheless, there are no treatments or vaccines available to cure this infection [4]. MAP transmission primarily occurs through the fecal-oral route [2], and it is estimated that more than half of dairy cattle herds in major dairy-producing countries are affected [5,6].

Although the molecular epidemiology of MAP has been documented in North America and Europe, the population-genetic framework of MAP isolates from the East Asian axis remains critically underrepresented, forming a genomic void in understanding its global genotypic variation. In South Korea, the reported prevalence has gradually increased [7,8]; this trend likely reflects enhanced diagnostic surveillance rather than a sudden expansion of MAP exposure. Consequently, high-resolution genomic data providing direct comparisons with global lineages remains lacking. Furthermore, the expanding epidemiological reach of MAP into wildlife reservoirs, such as native black goats, underscores the need for tools to trace interspecies transmission [9,10,11,12].

Whole genome sequencing (WGS) is a powerful tool, but its high cost and analytical complexity often limit the scalability for routine, large-scale national monitoring [13], especially in developing countries. Consequently, there is a critical need for cost-effective, high-throughput molecular typing methods that provide sufficient resolution for epidemiological investigations and to guide WGS-based follow-up studies. Traditional methods, such as IS*1311* polymerase chain reaction-restriction enzyme analysis (PCR-REA), provide broad lineage categorization (Type I and III, sheep-type; Type II, cattle-type; and B type, bison-type), but fail to delineate fine-scale clonal movements [14]. Mycobacterial interspersed repetitive unit-variable number tandem repeat (MIRU-VNTR) remains a global staple, and integration of multilocus short sequence repeats (MLSSRs) targeting hypervariable loci offers superior discriminatory power [15,16,17]. This combined approach enables the identification of subtype-level variation and partially bridges the data gap in East Asia, while remaining more feasible for routine use than WGS [18,19].

This study characterized 12 MAP isolates, including 9 bovine and 3 caprine (Korean black goat) strains recovered from 2021 to 2024, and compared them with 34 global reference strains from 6 continents using IS*1311* PCR–REA, MIRU-VNTR, and MLSSR typing. This study aimed to describe the population structure of Type II MAP circulating in South Korea within a global context, to illustrate the utility of MLSSR-integrated surveillance as a higher-resolution complement to MIRU-VNTR, and to provide a reference framework that can inform future WGS-based studies and control strategies for Johne’s disease.

## METHODS

### Sample preparation and fecal detection of MAP

Between 2021 and 2024, MAP detection was performed on the genomic DNA extracted from 1,056 individual fecal samples (approximately 22 animals per herd) collected from 37 herds across 8 provinces in South Korea. For DNA extraction, 2 g of feces was mixed with 35 mL of distilled water (DW), allowed to stand for 30 min; the upper 5 mL of the supernatant was used. The genomic DNA was extracted using a fecal microbe DNA extraction kit (Zymo Research, USA) according to the manufacturer’s protocol, and the DNA concentration was quantified using an ND–1000 Spectrophotometer (NanoDrop, USA). MAP positivity was confirmed by quantitative polymerase chain reaction (qPCR) targeting IS900 and ISMap02, as described elsewhere [8]. In addition, the genomic DNA samples of MAP were extracted from the tissues and feces of three farmed black goats from three herds across South Korea, provided by the National Institute of Animal Science (NIAS, Korea) [20].

### Bacterial isolation using the fecal decontamination procedure

Twelve Korean MAP isolates (9 from cattle and 3 from black goats) were obtained for genotyping. Among all qPCR-positive fecal samples, the MAP culture was attempted, and the isolates from animals in which culture was established were retained. Thus, the 12 Korean isolates analyzed in this study represent all available culture-positive cases with sufficient growth and DNA yield for downstream typing. The cattle isolates were obtained from PCR-positive fecal samples collected from four regions (Gyeonggi, Gangwon, Gyeongbuk, and Incheon). NIAS conducted passive surveillance to identify the MAP infections in clinical suspect cases, and three MAP strains were isolated from the feces of black goats in the Gyeongnam region exhibiting the clinical signs of Johne’s disease. After isolation and cultivation at NIAS, the genomic DNA of these isolates was provided for this study. Isolation and cultivation of MAP from fecal specimens were performed using the VersaTREK system (Thermo Scientific, USA) using a procedure described elsewhere [21]. After decontamination, the suspension was inoculated into VersaTREK Para-JEM broth supplemented with Para-JEM GS, AS, EYS, and Blue (Thermo Scientific) and incubated at 37°C in the VersaTREK instrument. After up to 8 weeks, instrument-positive cultures were inoculated (≤ 100 µL) onto 7H10 agar containing amphotericin, nalidixic acid, vancomycin, and Mycobactin J (2 mg/L, Allied Monitor, USA) and cultured for 4–6 weeks. The colonies were then grown in 10 mL Middlebrook 7H9 broth (Becton Dickinson Biosciences, USA) with 0.2% glycerol, 0.05% Tween 80, Mycobactin J, and 10% oleic-albumin-dextrose-catalase enrichment at 37°C with agitation for 6 weeks. The colonies were confirmed as MAP-positive by duplex real-time PCR using species-specific IS*900* and ISMap*02* primers.

### Genetic information of Korean and global isolates of MAP

For the 12 Korean isolates, the target loci were amplified by PCR and sequenced using a commercial facility (Macrogen, Korea) through standard Sanger sequencing. A comparative context was provided by retrieving 34 reference sequences from the National Center for Biotechnology Information (NCBI) database and grouping them according to the continent of origin: Asia (n = 14), Europe (n = 8), North America (n = 5), South America (n = 3), Africa (n = 2), and Oceania (n = 2). This study included MAP genomes for which all MIRU-VNTR and MLSSR target loci could be identified from the available assemblies, together with metadata specifying the geographic origin and host. Supplementary Table 1 lists the detailed information on these reference strains, including strain names and GenBank accession numbers.

### IS*1311* PCR-REA-based typing

Strain typing was performed on the Korean isolates using IS*1311* PCR-REA to differentiate between sheep (Type I and III), cattle (Type II), and bison types (B type). Briefly, a 223 bp region of the IS*1311* sequence was amplified with the primers M56 and M119 [22]. Differential analysis was then performed by electrophoresis on a 2% agarose gel after HinfI digestion of the amplified PCR products [23].

In contrast, the global MAP strains were classified through *in silico* analysis of the genomic data. Specifically, the genomic sequences were downloaded in FASTA format from the NCBI GenBank assemblies (Supplementary Table 1). The regions containing the informative IS*1311* sequences were selected and aligned using the bioinformatics software Unipro UGENE (v.49.0; Unipro, Russia) [24]. The strain types were then determined by identifying the specific polymorphism sites that distinguish among Type I, II, III, and B type MAP strains, according to the criteria described elsewhere [25].

### MIRU-VNTR and MLSSR typing

MIRU-VNTR typing was carried out with 12 Korean MAP isolates and 34 isolates retrieved from NCBI by using 8 MIRU-VNTR targets (292, X3, 25, 47, 3, 7, 10, and 32) [15]. The number of repeats in the PCR products of Korean MAP isolates was determined by analyzing the bands on a 2% agarose gel after PCR amplification [15]. For the 34 global reference strains, the corresponding alleles were characterized by *in silico* analysis to ensure comparability with the laboratory data. Specifically, each locus was curated manually by mapping the primer pairs [15] to the NCBI genome assemblies to determine the precise amplicon boundaries. The number of repeats was then calculated by subtracting the flanking-region length and dividing the result by the known repeat unit size, as specified in the MAC-INMV database. All *in silico* allele calls were visually verified to ensure data integrity; only the assemblies with complete data for all eight loci were retained for comparative clustering.

MLSSR typing of 12 Korean isolates was performed through PCR amplification followed by sequencing [16,26], and the repeat numbers at each SSR locus were determined. For 34 global strains, *in silico* MLSSR typing was performed by manually identifying the 11 SSR loci using the specified primer sets [16]. The primers were searched against each assembly to locate the target regions. The inter-primer sequences were then manually inspected, and the number of mono- or dinucleotide repeats was counted according to the MAC-INMV-SSR nomenclature. When an SSR locus could not be located unambiguously, or the repeat tract was truncated at a contig edge, that locus was recorded as missing for the corresponding strain. For MLSSR analysis, alleles with > 11 repeats were treated as distinct alleles and included as separate categories in the diversity calculations. Isolates without a matching MLSSR type in the MAC-INMV-SSR database were labeled “No Database” and considered novel. SNPs in the SSR flanking regions that did not alter the repeat counts were ignored for allele designation.

### Calculation of discriminatory power

The discriminatory power of the MIRU-VNTR and MLSSR typing methods was quantitatively evaluated using the discriminatory index (DI), as proposed by Hunter and Gaston [27]. The DI was calculated using the following equation:


$$DI=1-\left[\frac{1\;}{N(N-1)}\sum_{j=\;1}^{s}n{\;}_{j}\;(n{\;}_{j}\;-1)\right]$$

where *N* represents the total number of isolates in the typing scheme; *s* denotes the total number of distinct types identified by each typing method; and n*_j_* indicates the number of isolates belonging to the *j*^th^ type.

### Analysis of genetic relationship

The genetic relationships among the 46 strains were assessed by constructing a minimum spanning tree (MST) based on 11 SSR markers. The MST was generated in Python (version 3.11; Python Software Foundation, USA) using NetworkX and Matplotlib for graph operations and visualization. The nodes were linked according to shortest genetic distances, with node sizes proportional to the number of isolates and colors indicating the continent of origin.

### Statistical analysis

The associations between the host age and MAP prevalence in beef and dairy cattle were assessed. Table 1 lists the age-stratified prevalences (≤ 2, 3–4, 5–6, ≥ 7 years, and unknown). A Fisher’s exact test was used within each species to compare the distribution of MAP-positive animals across age categories. All analyses were conducted using GraphPad Prism version 9.4.0 (GraphPad Software, USA), with *p* < 0.05 considered significant.

**Table 1:** Prevalence and age distribution of *Mycobacterium avium* subsp. *paratuberculosis* infection in beef and dairy cattle herds in South Korea

Species	PCR	No. of positive samples/tested (%) Age distribution (yr)					No. of samples positive (%)
		≤ 2	3–4	5–6	≥ 7	Unknown	
Beef	IS*900*	70/347 (20.2)	-	-	2/79 (2.5)	-	72/719 (10.0)
	ISMap*02*	34/347 (9.8)	-	-	2/79 (2.5)	-	36/719 (5.0)
	Both	34/347 (9.8)	-	-	2/79 (2.5)	-	36/719 (5.0)
	Subtotal	34/347 (9.8)^*^	0/93 (0.0)	0/79 (0.0)	2/79 (2.5)	0/121 (0.0)	36/719 (5.0)
Dairy	IS*900*	14/115 (12.2)	8/106 (7.5)	16/44 (36.4)	3/21 (14.3)	-	41/337 (12.2)
	ISMap*02*	3/115 (2.6)	5/106 (4.7)	13/44 (29.5)	2/21 (9.5)	-	23/337 (6.8)
	Both	3/115 (2.6)	5/106 (4.7)	13/44 (29.5)	2/21 (9.5)	-	23/337 (6.8)
	Subtotal	3/115 (2.6)	5/106 (4.7)	13/44 (29.5)^*^	2/21 (9.5)	0/51 (0.0)	23/337 (6.8)
Total		37/462 (8.0)	5/199 (2.5)	13/123 (10.6)	4/100 (4.0)	0/172 (0.0)	59/1,056 (5.6)

PCR, polymerase chain reaction.

^*^Significant difference compared to other age groups within the same row (*p* < 0.05), “Both” indicates combined results of IS*900* and ISMap*02*, “Subtotal” indicates the sum of the counts within the respective subgroup.

## RESULTS

### Prevalence of MAP infection in Korean cattle herds

PCR amplification targeting IS*900* and ISMap*02* was conducted using feces collected from 719 cattle in 19 native cattle herds and 337 cows in 18 dairy herds between March 2021 and December 2024 (Table 1). The IS*900* gene was detected in 113 samples (10.7%), whereas the ISMap*02* gene was detected in 59 IS*900*-positive samples (5.6% of the total samples), which were considered as definite MAP positives (5.6%). Of the tested herds, 43.2% of the tested herds (16 out of 37 herds) tested positive for a MAP infection. Statistical analysis revealed significant differences in the age distribution of MAP-positive samples (Table 1). In beef cattle, the proportion of MAP-positive animals was highest in the ≤ 2-year group (34/347, 9.8%), which was significantly greater than in the older age groups (3–4, 5–6, and ≥ 7 years; *p* < 0.05). In contrast, in dairy cattle, the highest prevalence was observed in animals aged 5–6 years (13/44, 29.5%), which was significantly higher than in the other age categories (≤ 2, 3–4, and ≥ 7 years; *p* < 0.05). Specifically, no positive animals were detected among beef cattle aged 3–4 or 5–6 years. The 12 Korean isolates originated from Central (n = 4), Northeastern (n = 3), and Southeastern (n = 5) regions (Supplementary Fig. 1).

### Molecular typing of MAP Korean isolates

Genotyping of Korean MAP isolates was performed using IS*1311* PCR–REA, MIRU-VNTR, and MLSSR (Table 2). All isolates were Type II by IS*1311* PCR–REA. MIRU-VNTR analysis revealed a single type, INMV 2, across all isolates. MLSSR typing identified 11 SSR loci in 12 MAP isolates, with MLSSR 18 in 4 isolates (33.3%) and MLSSR 55 in 1 isolate (8.3%). The black goat isolate belonged to MLSSR 55, whereas 7 isolates could not be assigned to known MLSSR types. Among the 11 MLSSR loci, loci 2, 8, and 9 were polymorphic, with 2 to 4 alleles, whereas the others were monomorphic. Locus 2 had the highest allelic diversity (DI = 0.742), and loci 8 and 9 had DI values of 0.409. The overall DI value of the MLSSR typing in the 12 Korean isolates was 0.864.

**Table 2:** Geographic distribution of IS*1311* PCR-REA genotypes identified in MAP fecal DNA and MAP isolates obtained from cattle and black goats in South Korea

Region	Sample/Name	No. of copies of MIRU-VNTR								INMV group	IS*1311* PCR-REA	MLSSR locus											MLSSR type
		292	X3	25	47	3	7	10	32			1	2	3	4	5	6	7	8	9	10	11	
Gangwon	MAP_GW63	3	2	3	3	2	2	2	8	INMV2	C strain (Type II)	7	11	5	5	5	5	5	6	5	5	5	MLSSR 18
	MAP_GW79	3	2	3	3	2	2	2	8	INMV2	C strain (Type II)	7	11	5	5	5	5	5	6	5	5	5	MLSSR 18
	MAP_GW109	3	2	3	3	2	2	2	8	INMV2	C strain (Type II)	7	10	5	5	5	5	5	6	5	5	5	No Database
Incheon	MAP_IC07	3	2	3	3	2	2	2	8	INMV2	C strain (Type II)	7	11	5	5	5	5	5	6	5	5	5	MLSSR 18
	MAP_IC92	3	2	3	3	2	2	2	8	INMV2	C strain (Type II)	7	11	5	5	5	5	5	6	5	5	5	MLSSR 18
Gyeonggi	MAP_GG86	3	2	3	3	2	2	2	8	INMV2	C strain (Type II)	7	10	5	5	5	5	5	6	5	5	5	No Database
	MAP_GG33	3	2	3	3	2	2	2	8	INMV2	C strain (Type II)	7	10	5	5	5	5	5	6	5	5	5	No Database
Gyeongbuk	MAP_GB31	3	2	3	3	2	2	2	8	INMV2	C strain (Type II)	7	> 11	5	5	5	5	5	6	5	5	5	No Database
	MAP_GB81	3	2	3	3	2	2	2	8	INMV2	C strain (Type II)	7	> 11	5	5	5	5	5	6	5	5	5	No Database
Gyeongnam	MAP_15-25^a^	3	2	3	3	2	2	2	8	INMV2	C strain (Type II)	7	10	5	5	5	5	5	5	4	5	5	No Database
	MAP_19-13^a^	3	2	3	3	2	2	2	8	INMV2	C strain (Type II)	7	11	5	5	5	5	5	5	4	5	5	No Database
	MAP_20-12^a^	3	2	3	3	2	2	2	8	INMV2	C strain (Type II)	7	> 11	5	5	5	5	5	5	4	5	5	MLSSR 55

PCR-REA, polymerase chain reaction-restriction enzyme analysis; MAP, *Mycobacterium avium* subsp. *paratuberculosis*; MIRU-VNTR, mycobacterial interspersed repetitive unit-variable number tandem repeat; MLSSR, multilocus short sequence repeat.

^a^Black goat samples used in this study, “> 11” indicates alleles with more than 11 repeats, “No Database” indicates novel MLSSR types not present in the MAC-INMV-SSR database.

### Distribution of MAP genotypes of global strains

MAP sequences from NCBI were grouped by continent to compare the genotype distributions. The genotypes were determined using established genomic features that distinguish Type I from Type II strains, including the 11-bp insertion and type-specific loci [25]. Among the 46 genomes from six continents, 38 were Type II and 3 were B type. The remaining 5 were S-type strains, including three Type I and two Type III isolates (Table 3). Type II strains were identified on 5 continents, whereas B type strains were detected only in Asia. The Type I strains were identified on 2 continents but with distinct subtype distributions: Type I in North America and Oceania, and Type III in Europe and North America (Table 3).

**Table 3:** Continent-level distribution of IS*1311* PCR-REA genotypes in MAP isolates worldwide

Continent	S strain (Type I)	S strain (Type III)	B strain (B type)	C strain (Type II)
Asia (n = 26)	-	-	3	23
Europe (n = 8)	-	1	-	7
North America (n = 5)	1	1	-	3
South America (n = 3)	-	-	-	3
Africa (n = 2)	-	-	-	2
Oceania (n = 2)	2	-	-	-

PCR-REA, polymerase chain reaction-restriction enzyme analysis; MAP, *Mycobacterium avium* subsp. *paratuberculosis*.

Using MIRU-VNTR, the 46 MAP isolates were classified into 14 subtypes (Table 4). INMV 2 was the most prevalent type, accounting for 60.9% (n = 28) of all isolates. The distribution of MIRU-VNTR subtypes showed distinct regional patterns. In Asia, 3 MIRU-VNTR loci (MIRU 292, VNTR 25, and VNTR 7) were polymorphic, with 3 patterns in VNTR 25 and 2 genotypes in MIRU 292 and VNTR 7. In Europe, all loci except MIRU X3 and VNTR 32 were polymorphic, whereas in North America, all loci except VNTR 47 were polymorphic. In South America and Africa, only INMV 2 was identified, whereas only INMV 72 was identified in Oceania. MIRU 292 showed the highest genetic diversity. The allelic diversity values were 0.428 for MIRU 292, 0.159 for MIRU X3, 0.334 for VNTR 25, 0.083 for VNTR 47, 0.194 for VNTR 3, 0.258 for VNTR 7, 0.227 for VNTR 10, and 0.084 for VNTR 32. The overall genetic diversity index was 0.630.

**Table 4:** MIRU-VNTR profiles of *Mycobacterium avium* subsp. *paratuberculosis* isolates at the continent-level

Continent	INMV group	No. of isolates (%)	No. of copies of MIRU-VNTR								Numerical code
			292	X3	25	47	3	7	10	32	
Asia (n = 26)	INMV 2	20 (43.5)	3	2	3	3	2	2	2	8	32332228
	INMV 6	1 (2.2)	3	2	3	3	2	1	2	8	32332128
	INMV 39	2 (4.3)	3	2	5	3	2	2	2	8	32532228
	INMV 68	2 (4.3)	2	2	5	3	2	2	2	8	22532228
	INMV 149	1 (2.2)	2	2	4	3	2	2	2	8	22432228
Europe (n = 8)	INMV 1	1 (2.2)	4	2	3	3	2	2	2	8	42332228
	INMV 2	2 (4.3)	3	2	3	3	2	2	2	8	32332228
	INMV 6	1 (2.2)	3	2	3	3	2	1	2	8	32332128
	INMV 13	1 (2.2)	2	2	3	3	2	2	2	8	22332228
	INMV 33	2 (4.3)	3	2	5	2	2	2	2	8	32522228
	INMV 266	1 (2.2)	4	2	1	3	1	1	1	8	42131118
North America (n = 5)	INMV 2	1 (2.2)	3	2	3	3	2	2	2	8	32332228
	INMV 70	1 (2.2)	7	1	3	3	1	1	1	8	71331118
	INMV 71	1 (2.2)	5	1	1	3	1	1	1	8	51131118
	No DB_1	1 (2.2)	2	2	3	3	2	2	1	2	22332212
	No DB_2	1 (2.2)	2	2	3	3	2	2	2	7	22332227
South America (n = 3)	INMV 2	3 (6.5)	3	2	3	3	2	2	2	8	32332228
Africa (n = 2)	INMV 2	2 (4.3)	3	2	3	3	2	2	2	8	32332228
Oceania (n = 2)	INMV 72	2 (4.3)	4	1	3	3	1	1	1	8	41331118

MIRU-VNTR, mycobacterial interspersed repetitive unit-variable number tandem repeat.

Eleven SSR loci were characterized in 46 MAP global isolates. Thirty-one MLSSR subtypes were identified among these 46 isolates (Table 5). The most prevalent subtype was MLSSR type 9, which was identified in 6 isolates (13%). Of the 31 MLSSR subtypes detected in this study, most were unique to a single isolate, and loci 4 and 5 were not polymorphic, remaining invariant across all isolates. Locus 8 had the highest diversity with a DI value of 0.723, while the allelic diversity of loci 1, 2, 3, 6, 7, 9, 10, and 11 were 0.441, 0.571, 0.198, 0.507, 0.428, 0.511, 0.125, and 0.198, respectively. In the combined global dataset, MLSSR typing identified 31 subtypes with an overall DI of 0.973. This high level of allelic diversity enabled the identification of identical MLSSR genotypes in isolates originating from different continents (Table 5). Africa and Oceania generally showed distinctive types, but three intercontinental clusters with shared MLSSR profiles were observed: 1) Asia type 3 was identical to Europe type 16 and South America type 26; 2) a shared profile was identified among Asia type 6, Europe type 13, and North America type 24; and 3) Asia type 7 matched North America type 20.

**Table 5:** MLSSR profiles of *Mycobacterium avium* subsp. *paratuberculosis* isolates at the continent-level

Continent	MLSSR type	No. of isolates (%)	MLSSR locus										
			1	2	3	4	5	6	7	8	9	10	11
Asia (n = 26)	1	1 (2.2)	> 11	9	5	5	5	5	5	5	5	5	5
	2	3 (6.5)	7	> 11	5	5	5	4	4	4	4	5	5
	3	2 (4.3)	7	> 11	5	5	5	4	5	4	4	5	5
	4	1 (2.2)	9	10	5	5	5	5	5	5	5	5	5
	5	2 (4.3)	7	9	5	5	5	4	4	4	4	5	5
	6	1 (2.2)	> 11	10	5	5	5	5	5	5	5	5	5
	7	3 (6.5)	7	> 11	5	5	5	5	5	5	5	5	5
	8	1 (2.2)	7	10	5	5	5	5	5	5	4	5	5
	9^a^	6 (13)	7	> 11	5	5	5	5	5	6	5	5	5
	10^a^	3 (6.5)	7	10	5	5	5	5	5	6	5	5	5
	11^a^	2 (4.3)	7	> 11	5	5	5	5	5	5	4	5	5
	12^a^	1 (2.2)	7	10	5	5	5	5	5	5	4	5	5
Europe (n = 8)	13	1 (2.2)	> 11	10	5	5	5	5	5	5	5	5	5
	14	1 (2.2)	7	9	5	5	5	4	6	4	4	5	5
	15	1 (2.2)	7	10	5	5	5	4	6	4	5	5	5
	16	2 (4.3)	7	> 11	5	5	5	4	5	4	4	5	5
	17	1 (2.2)	7	9	5	5	5	4	7	4	4	4	5
	18	1 (2.2)	7	> 11	4	5	5^b^	4	4	3	4	4	4
	19	1 (2.2)	10	> 11	5	5	5	4	5	4	5	5	5
North America (n = 5)	20	1 (2.2)	7	> 11	5	5	5	5	5	5	5	5	5
	21	1 (2.2)	10	> 11	5	5	5	5	5	5	5	5	5
	22	1 (2.2)	5	> 11	4	5	5^b^	4	5	3	4	5^b^	4
	23	1 (2.2)	7	10	4	5	5^b^	4	4	3	4	5^b^	4
	24	1 (2.2)	> 11	10	5	5	5	5	5	5	5	5	5
South America (n = 3)	25	1 (2.2)	7	10	5	5	5	4	4	4	4	5	5
	26	1 (2.2)	7	> 11	5	5	5	4	5	4	4	5	5
	27	1 (2.2)	7	> 11	5	5	5	4	5	4	5	5	5
Africa (n = 2)	28	1 (2.2)	> 11	> 11	5	5	5	5	5	5	5	5	5
	29	1 (2.2)	> 11	10	5	5	5	5	5	4	4	4	5
Oceania (n = 2)	30	1 (2.2)	8	> 11	4	5	5^b^	4	5	3	4	5^b^	4
	31	1 (2.2)	10	> 11	4	5	5^b^	4	5	3	4	5^b^	4

MLSSR, multilocus short sequence repeat.

^a^Domestic isolates in this study, ^b^single nucleotide polymorphisms, “> 11” indicates alleles with more than 11 repeats.

### Genetic relationship of isolates by continent

**Fig. 1:**
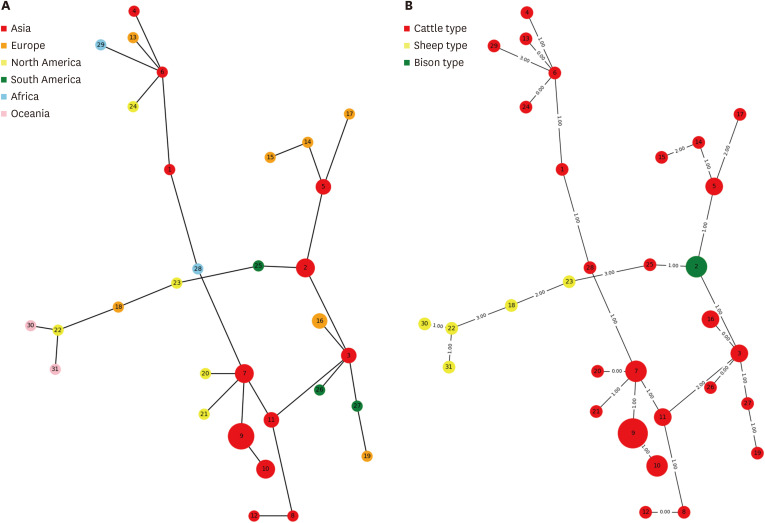
Minimum spanning tree based on the MLSSR genotypes among *Mycobacterium avium* subsp. *paratuberculosis* isolates from different continents globally. Each genotype is represented by a pie chart, with the chart size reflecting the number of strains; the colors indicate the distribution of strains by continent (A) or by IS*1311* polymerase chain reaction-restriction enzyme analysis type (B). The numbers on the pie chart indicate the MLSSR type. MLSSR, multilocus short sequence repeat.

The genetic relatedness of the 46 isolates was illustrated using the MST based on 11 SSR loci (Fig. 1). The predominant genotypes were types 2, 7, 9, and 10; type 2 belonged to the B type genotype, whereas types 7, 9, and 10 belonged to Type II. The South American and African isolates clustered with the Type II strains. Although Type II predominated in Europe and North America, some MLSSR types also included Type I isolates, and all Oceania isolates clustered with the Type I strains. MLSSR types 6, 13, and 24, including isolates 3, 16, and 26, were obtained from different continents. These strains shared identical MLSSR profiles and were classified as Type II, indicating close relatedness at the subtyping level.

## DISCUSSION

The prevalence of MAP infection in South Korea and the genetic comparison of domestic isolates with global strains were investigated to identify control measures for Johne’s disease, which causes substantial economic losses in cattle farms. The MAP-positive rates in this study increased at the herd and individual levels compared with previous reports [7,8], regardless of the farm type, suggesting ongoing spread of MAP across Korean cattle farms. The MAP-positive status was determined using MAP-specific genomic detection in fecal samples, as described previously [28,29,30].

Unlike previous studies [7,8,30], this study revealed distinct age-related patterns. In beef herds, cattle ≤ 2 years old had the highest infection rate, whereas in dairy herds, the prevalence peaked in animals aged 5–6 years. These differences may reflect production cycles and management practices. In beef herds, the high prevalence in younger cattle may result from prolonged cow–calf contact and shorter rearing periods before slaughter [31]. In dairy herds, the peak prevalence in older cattle likely reflects the long incubation period of MAP combined with management practices, environmental exposure, and cumulative physiological stress [32].

The genetic epidemiological characteristics of the isolates were analyzed using IS*1311* PCR–REA, MIRU-VNTR, and MLSSR, together with the international isolates from NCBI. All domestic strains were classified as Type II by IS*1311* PCR-REA, consistent with reports that Type II is common worldwide [33]. Furthermore, Type II MAP was identified in Korean native black goats, suggesting that this lineage infects multiple domestic ruminant species. Together with previous reports of MAP infection in wild boars and livestock such as cattle, goats, and elk [8,34], these findings suggest that Type II MAP is circulating in various hosts in South Korea. On the other hand, further high-resolution phylogenomic studies with stronger epidemiologic linkages are required to establish specific transmission dynamics and the direction of spread.

Fourteen INMV types were observed by MIRU-VNTR, with all Korean isolates belonging to INMV 2. In Asia, 20 Type II isolates (43.5%) were INMV 2, whereas the other isolates were INMV 6, 39, 68, and 149. INMV 2 and 6 are the most prevalent types worldwide [35,36,37,38], and INMV 39, 68, and 149 represent genotypes not reported elsewhere. The European isolates included INMV 13 and 33, described in Argentina [38,39], as well as globally distributed INMV 1, 2, and 6 [35,40]. The INMV 266 type was the only type that had not been reported. In North America, South America, and Africa, INMV 2 was the predominant type, while INMV 70 and 71 were confirmed to be novel types in the USA [41,42]. The dominance of a single INMV type in South Korea does not necessarily imply a single introduction, underscoring the need for high-resolution typing methods for accurate surveillance [43].

Among the 12 Korean isolates, only a subset could be assigned to known MLSSR types (MLSSR 18 and 55), whereas the remaining isolates represented novel MLSSR genotypes not present in the current database. The SSR loci 3, 4, 5, 10, and 11 were not variable for the MAP isolates from worldwide, as reported elsewhere [15,44]. The most variable SSR marker for the isolates was loci 2 and 8 in South Korea and worldwide, respectively, which revealed 4 different alleles. Despite the concerns regarding SSR locus 2 stability [18,45], these results highlight its high discriminatory power in combination with other loci and underscore the need to interpret the variation at this locus with caution.

The MLSSR typing results were used to generate an MST illustrating clonal distances among isolates from South Korea and other continents. In this MST, the Type I and II isolates formed distinct clusters, whereas Type II and B type overlapped, consistent with previous MST analyses [30], supporting the close relatedness between B type and Type II MAP [46,47]. Nevertheless, higher-resolution genomics is still required for detailed phylogenetic inference.

Despite the limitations regarding the isolated numbers and DNA quality, these findings effectively reflect the epidemiological landscape of MAP both nationwide and globally. Specifically, the 12 domestic MAP isolates newly characterized in this study expand the known genomic diversity of Type II MAP in Korea by identifying the globally common genotypes and previously unreported, novel MLSSR types. Building on previous research [30], this study reinforces the evidence of MAP strains circulating among Korean ruminants by identifying infections in black goats. The observed allelic diversity (DI = 0.973) indicates that MLSSR provides high discriminatory power for subtyping cattle-type MAP and for identifying the clusters spanning multiple continents. These findings support the use of MLSSR as a primary screening tool to identify high-priority clusters for subsequent WGS-based analysis. Although this approach does not provide genome-wide resolution or reconstruct detailed transmission pathways, it narrows the genomic information gap in East Asia. This approach also offers a preliminary, East Asia-inclusive comparative framework that can be integrated into future WGS-based surveillance and control efforts for Johne’s disease.

## SUPPLEMENTARY MATERIALS

Supplementary Table 1 Summary of 34 *Mycobacterium avium* subsp. *paratuberculosis* reference genomes categorized by continent

Supplementary Fig. 1 Geographic mapping of MAP isolates in South Korea. The 12 isolates were collected from five provinces, which were categorized into three major geographical regions: Central (Red, Incheon [IC]; Yellow, Gyeonggi [GG]), Northeastern (Green, Gangwon [GW]), and Southeastern (Pink, Gyeongbuk [GB]; Orange, Gyeongnam [GN]). The colors indicate the respective provinces from which the isolates were obtained.
